# Supplementary material for: Evidence for late-glacial oceanic carbon redistribution and discharge from the Pacific Southern Ocean
Source: Nat Commun. 2022 Nov 11;13:6250. doi: 10.1038/s41467-022-33753-4 (PMC9652385; doi:10.1038/s41467-022-33753-4)
Supplement: Supplementary file 1 — Supplementary Information [file 41467_2022_33753_MOESM1_ESM.pdf]

Supplementary Information

Supplementary Figures

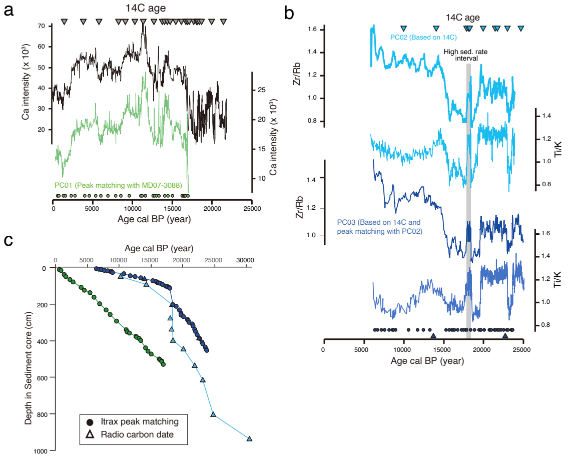

Supplementary Figure 1. The age model of sediment cores. a Peak matching of variation in Ca intensity between well-aged core MD07-3088 and PC01. **b** Peak matching of variation in Zr/Rb ratio between PC02, aged by 14C-age dating, and PC03. The variations in Ti/K ratio in each core are also shown. **c** Plots of depth in core (cm) vs age (ya). Tie-points of peak matching and radio carbon dating is shown.

a. PS97/114 (Mutiple core)

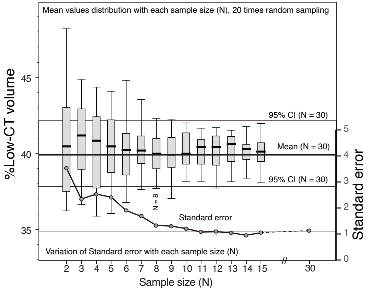

b. PS97/129 (Mutiple core)

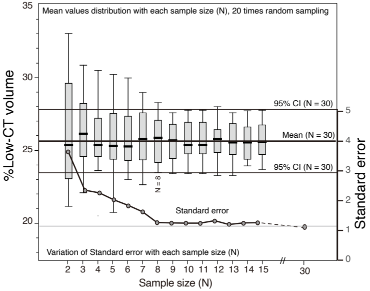

Supplementary Figure 2. Changes in mean %Low-CT number calcite volume distribution and standard error with random selected different sample sizes. Test samples were obtained from multiple cores, **a** high %Low-CT number calcite volume (relatively dissolved) and **b** low %Low-CT number calcite volume (relatively preserved) samples, respectively. Mean values distribution and standard error derived from each sample size are compared with the results of largest sample size (N = 30).

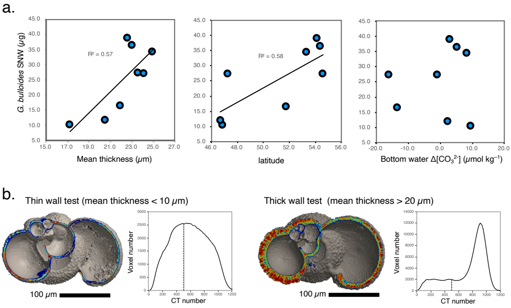

Supplementary Figure 3. Controlling factor of foraminiferal test weight and the effect of test wall thickness to tomography data. a Plots of Size normalized weight (SNW: µg) against mean thickness of outermost chamber wall (µm) of *G. bulloides* tests, latitude of sampling sites of multiple cores, and bottom water carbonate saturation state ($\Delta[CO_3^{2-}]$) at sampling sites of multiple cores. Regression lines are shown. **b** Different types of *G. bulloides* test found in the sediment samples. The test samples with thin outer chamber wall as shown in the figure are excluded from dissolution intensity measurement.

Supplementary Table 1, Bottom water carbonate saturation state of sea floor sediment samples

Sample	Latitude	Longitude	Depth (m)	$\Delta[CO_3^{2-}]$ (μmolkg^{-1})	$[CO_3^{2-}]$ (μmolkg^{-1})
MR16-09_MC01	46°04′ S	75°41′ W	1537	9.46	66.43
MR16-09_MC02	46°04′ S	76°32′ W	2787	2.42	75.74
MR16-09_PL03	46°24′ S	77°19′ W	3074	-1.01	74.57
MR16-09_MC04	50°48′ S	79°07′ W	3851	-13.42	77.26
PS97/27	54°38′ S	74°61′ W	2363	5.12	72.50
PS97/114	54°58′ S	76°65′ W	3869	-16.11	74.92
PS97/122	54°10′ S	74°92′ W	2558	2.87	72.95
PS97/199	53°32′ S	75°21′ W	1870	8.30	69.31
