# Supplementary material for: Evidence for late-glacial oceanic carbon redistribution and discharge from the Pacific Southern Ocean
Source: Nat Commun. 2022 Nov 11;13:6250. doi: 10.1038/s41467-022-33753-4 (PMC9652385; doi:10.1038/s41467-022-33753-4)
Supplement: Supplementary file 3 — Description of Additional Supplementary Files [file 41467_2022_33753_MOESM3_ESM.pdf]

File name: Supplementary Data 1

Description: Data of Multiple core samples

File name: Supplementary Data 2

Description: Data of Piston core samples

File name: Supplementary Data 3

Description: Data of stable isotope in core PC03.

File name: Supplementary Data 4

Description: Data of XRF in core PC02 and PC03.

File name: Supplementary Data 5

Description: Age models of piston core.
